# PLEKHG2 Promotes NSCLC Cell Growth by Increasing Glycolysis via Activated PI3K/AKT Pathway

**DOI:** 10.7150/jca.88857

**Published:** 2023-10-30

**Authors:** Yang Xia, Xinyu Feng, Yunye Ning, Wei Zhang, Zhenli Hu, Qianqian Chen, Jun Wang, Hao Qin, Yang Lu, Yuchao Dong

**Affiliations:** 1 Department of Respiratory and Critical Care Medicine, the First Affiliated Hospital of Naval Medical University, Shanghai, People's Republic of China.; 2 Department of Cardiology, Seventh People's Hospital of Shanghai University of Traditional Chinese Medicine, Shanghai, China.

**Keywords:** PLEKHG2, NSCLC, glycolysis, PI3K/AKT pathway

## Abstract

**Purpose:** PLEKHG2 is a member of the diffuse B-cell lymphoma family. The function of PLEKHG2 in NSCLC was still unclear. This study aimed to investigate the relationship between the upregulated expression of PLEKHG2 and the prognosis of NSCLC and to revealed its mechanisms.

**Materials and methods:** The expression of PLEKHG2 in NSCLC patients and its relationship with prognosis were first determined by analyzing public databases. Validation was performed in NSCLC cell lines and patient`s tumor tissues. PLEKHG2-silenced H1299 cells and PLEKHG2 overexpressing PC9 cells were constructed and used to validate its function. Glycolysis was evaluated by assaying cellular metabolites, glucose uptake and the expression levels of biomarkers of glycolysis. The relationship of PLEKHG2 and the PI3K/Akt pathway was demonstrated by small molecule inhibitors. The function of PLEKHG2 was evaluated *in vivo* by a H1299 cell derived xenograft (CDX) model.

**Results:** PLEKEHG2 was highly expressed in NSCLC tissues and associated with poor prognosis. In PLEKHG2 knockdown H1299 cells, ATP and lactate production and glucose uptake were significantly inhibited. The opposite results were observed in PC9 cells with PLEKHG2 overexpression. The increased glycolysis following PLEKHG2 overexpression was abolished by adding the PI3K/AKT pathway inhibitor LY294002, suggesting that PLEKHG2 promotes glycolysis in NSCLC cells via activation of the PI3K/AKT pathway. Finally, we found that PLEKHG2 knockdown inhibited the tumor growth in the H1299 CDX model.

**Conclusion:** PLEKHG2 contributed to NSCLC development by promoting glycolysis via activation of the PI3K/AKT pathway. PLEKHG2 was a potential therapeutic target and biomarker for poor prognosis of NSCLC.

## 1. Introduction

With the gradual improvement of medical care, human lifespan and quality of life have been greatly improved. However, cancer still threatens human health and brings long-term economic and personal suffering to patients. According to Global Cancer Statistics from 2020, an estimated 19.3 million new cancer cases and 10 million cancer deaths occurred in 2020. Lung cancer is the second most prevalent cancer worldwide, with an estimated 2.2 million new cases (11.4%) and 1.8 million deaths (18%) anually^1^. Non-small cell lung cancer (NSCLC) accounts for approximately 85% of lung cancer cases^2^, and is often diagnosed at advanced stages where patients have poor prognoses^3^, ^4^. Over the past 20 years, various treatments including chemotherapy, targeted therapy, and immunotherapy have proven effective for some advanced NSCLC patients^5^. However, the overall survival (OS) and cure rates of NSCLC remain low, with a 5‑year survival rate of between 2% and 13% in patients with metastatic disease^6^. Therefore, exploring the in-depth molecular mechanisms of NSCLC development to identify potential targets for novel therapeutic strategies to improve survival is urgently needed.

Since the discovery of the Warburg effect in the 1920s, glycolysis has been revealed to be the most reliable metabolic hallmark of cancer cells^7^. The rate of ATP production from glycolysis is faster than mitochondrial respiration, even though it is not as efficient. Therefore, glycolysis is often used to generate the ATP needed to sustain the exaggerated growth of tumors^8^, ^9^. The lactate produced by tumor cell glycolysis creates an acidic environment that breaks down the extracellular matrix, facilitating the invasion and migration of tumor cells^10^. Although glycolysis has been used to detect various cancers, its causal relationship with cancer progression remains unclear.

Pleckstrin Homology And RhoGEF Domain Containing G2 (PLEKHG2) is a member of the diffuse B-cell lymphoma (Dbl) family that contains a highly conserved Dbl homology (DH) structural domain^11^. PLEKHG2 were mainly detected in nucleoplasm and Cytosol. In 2013, Runne et al. first reported that PLEKHG2 contributed to Jurkat T cell migration by promoting the heterotrimeric G protein βγ^12^, suggesting that PLEKHG2 may be associated with poor prognosis in leukemia. Subsequently, Katsuya et al. also demonstrated that PLEKHG2 affected cell morphology via the same mechanism^13^. However, in a subsequent study, Masashi et al. found that the interaction between PLEKHG2 and ABL1 inhibited the growth of HEK293 cells 14. Thus, PLEKHG2 may be associated with tumorigenesis and progression, but its function in NSCLC remains unknown.

In this study we confirmed that PLEKGH2 was highly expressed in tumor tissues of NSCLC patients and correlated with poor prognosis. Then the function of PLEKHG2 was investigated in vivo and in vitro. We found that PLEKHG2 promoted NSCLC progression by enhancing glycolysis through activating the PI3K/AKT pathway.

## 2. Materials and methods

### 2.1 Bioinformatics and statistical analyses

Bioinformatics analysis was conducted using datasets from The Cancer Genome Atlas (TCGA, https://cancergenome.nih.gov/) and Gene Expression Omnibus (GEO, https://www.ncbi.nlm.nih.gov/geo/). PLEKHG2 expression was analyzed in RNA-seq data from TCGA-lung adenocarcinoma (LUAD) dataset, which contains 526 NSCLC cases and 59 normal lung samples. All TCGA data were analyzed using R 4.0.3 software, and then were further processed using SPSS 20.0 software (IBM, Armonk, NY, USA). Gene array expression profile data from GSE50081 were divided into two groups according to the median PLEKHG2 expression level: 91 overexpression and 90 reduced expression cases. Then a survival curve was constructed via the Kaplan-Meier method using the Survfit package of R.

### 2.2 Gene set enrichment analysis (GSEA)

Associations between PLEKHG2 and the biological signaling pathway gene set were analyzed using GSEA (v2.2, http://www.broad.mit.edu/gsea/). We also analyzed the TCGA-LUAD dataset to compare differential gene expression between the PLEKHG2-positive and -negative groups using the MSigDB C2 CP collection (canonical pathways gene set). Default settings were used, and thresholds for significance were determined by permutation analysis (1000 permutations). A gene set was considered significantly enriched when the False Discovery Rate (FDR) score was less than 0.25.

### 2.3 Immunohistochemistry (IHC)

Tissue biopsy samples from 5 patients with early-stage lung adenocarcinoma who received surgical treatment in December 2021 were used to perform IHC assays. Patients enrolled must be diagnosed as stage I according to the AJCC/UICC 8th edition lung cancer staging criteria and have been confirmed by the Department of Pathology to have both tumor and paraneoplastic tissue stored with biopsy samples that have been collected. The clinical characteristics of the patients for IHC were shown in Supplemental Table 3. Paired tumor and corresponding paracancerous tissues collected from the First Affiliated Hospital of Naval Medical University Tumors were extracted and fixed in 10% neutral buffered formalin for 24 h, embedded in paraffin, and sliced at 3 μm thickness. The sections were deparaffinized, rehydrated, dewaxed, and graded debenzolization. Anti-PLEKHG2 antibody (ab180156, Abcam, Cambridge, UK; 1:500) was used to detect PLEKHG2 expression. PLEKHG2 expression levels were confirmed by two professional staff of the Pathology Department of the First Affiliated Hospital of Naval Medical University.

### 2.4 Cell culture and reagents

One normal human bronchial epithelial cell line (16HBE) and four NSCLC cell lines (A549, H1299, H358, and PC9) were purchased from the American Type Culture Collection (ATCC; Manassas, VA, USA) and cultured according to standard protocols. A549 and PC9 cells were cultured in Dulbecco's modified Eagle's medium (DMEM; Hyclone, Logan, UT, USA). H1299, H358, and 16HBE cells were cultured in RPMI 1640 (Hyclone). All media were supplemented with 10% fetal bovine serum (Gibco, Waltham, MA, USA) and 1% penicillin/streptomycin (Solarbio, Beijing, China) Cells were maintained under an atmosphere containing 5% CO2 at 37°C. The PI3K inhibitor LY294002 (MedChemExpress, Monmouth Junction, NJ, USA; 10 μM) and the glucose uptake inhibitor Phloretin (150 μM, MedChemExpress) were used to treat cells for 24 h.

### 2.5 Quantitative real-time PCR (qPCR)

Total RNA was extracted from cells using TRIzol reagent (Invitrogen, Waltham, MA, USA). cDNA was reverse transcribed using M-MLV Reverse Transcriptase (Fermentas, Waltham, MA, USA), and qPCR was performed using a SYBR Green PCR kit (Thermo Fisher Scientific, Waltham, MA, USA) and detected using an ABI-7300 real-time detector (Applied Biosystems, Waltham, MA, USA) in accordance with the manufacturer's instructions. The qPCR primers used in this study are shown in Supplemental Table 1. Relative mRNA expression in each cell line was calculated by the 2-ΔΔCt method.

### 2.6 Western blot analysis

For western blot analysis, cell lysates were prepared from cell lines using a RIPA lysis buffer kit (Beyotime, Shanghai, China), and then protein concentrations were detected using the BCA assay (Beyotime). Next, 30 μg of each sample was separated by 10% sodium dodecyl sulfate polyacrylamide gel electrophoresis, and then transferred onto nitrocellulose membranes (Millipore, Burlington, MA, USA). The blots were blocked with 5% bovine serum albumin in TBST for 1 h at room temperature and incubated overnight with the following primary antibodies: anti-PLEKHG2 (ab180156, Abcam; 1:1000), anti-AKT (#9272, Cell Signaling Technology, Danvers, MA, USA; 1:1000), anti-p-AKT (#4060, Cell Signaling Technology; 1:2000), anti-HK2 (ab209847, Abcam; 1:1000), anti-GLUT1 (ab115730, Abcam; 1:5000), and anti-GAPDH (#5174, Cell Signaling Technology; 1:2000). Following three washes with TBST, the membrane was incubated with secondary antibodies for 1 h at 37℃, followed by three washes with TBST buffer. Immunoreactive bands were visualized using the Tanon High-sig ECL kit (Tanon Science and Technology Co., Ltd., Shanghai, China) and analyzed with a Vilber Fusion Fx5 Spectra (Vilber Lourmat, Marne La Vallée, France).

### 2.7 PLEKHG2 knockdown and overexpression

Two small hairpin RNAs targeting PLEKHG2 were designed as follows: 5′-GCGCATTCTCAAGTACCAT-3′ (shPLEKHG2-1) and 5′-CCCATGACATTCCCAAGTT-3′ (shPLEKHG2-2); a scrambled shRNA was applied as the negative control. shRNAs were cloned into pLKO.1 (Addgene, Watertown, MA, USA). Full-length PLEKHG2 cDNA was inserted into pLVX-Puro (Clontech, Mountain View, CA, USA). To prepare lentiviral particles, 293T cells were transfected with the viral plasmids psPAX2 and pMD2G using Lipofectamine 2000 (Invitrogen) following to the manufacturer's instructions. H1299 cells were transduced with lentivirus encoding shRNAs, and PC9 cells were transduced with lentivirus encoding the PLEKHG2 overexpression vector. PLEKHG2 expression was evaluated by qPCR and WB. PLEKHG2 knockdown (shPLEKHG2-1, shPLEKHG2-2) and overexpression (oePLEKHG2) stable cell lines were selected by 2.5 μg/mL puromycin (Acros Organics, Geel, Belgium) and used for further experiments.

### 2.8 Glucose uptake assay and measuring lactate and ATP production

The NSCLC cell lines H1299 and PC9 with or without lentiviral transduction were used to compare glucose uptake and lactate and ATP generation. Lactate (A019-1-1) and ATP (A095-1-1) were detected with commercial assay kits (JianCheng Bioengineering Institute, Nanjing, China) in accordance with the manufacturer's instructions. Briefly, 1×106 cell of each group were extracted to measure lactate and ATP at OD530 and OD636, respectively. Glucose uptake was detected using the 2NBDG Glucose Uptake Assay Kit (BioVision, Milpitas, CA, USA). The 2NDBG fluorescence intensity was measured using an Accuri C6 (BD Biosciences, Franklin Lakes, NJ, USA), and results were analyzed with FolwJo v10.0.7 software (FlowJo LLC, Ashland, OR, USA).

### 2.9 Tumor xenografts

Twelve male BALB/c nude mice (4-6 weeks old, 18-20 g) were obtain from SLAC Laboratory Animal Co., Ltd. (Shanghai, China) and were bred in a specific pathogen-free laboratory at Changhai Hospital with a 12 h:12 h light/dark cycle and ad libitum access to food and water. Animal handling and experimental procedures were approved by the Animal Experimental Ethics Committee of Changhai Hospital. The nude mice were randomly divided into the negative control (NC) and shPLEKHG2 groups (n=6 per group). H1299 cells transduced with shNC or shPLEKHG2 were injected subcutaneously into the left axilla, with 5×10^5^ cells per mouse. Once tumors became palpable, xenografts were measured with a caliper every 3 d. The volume of xenografts was calculated using the formula: V (mm3) = ½ × length × width^2^. All mice were euthanized 33 d after inoculation, at which time the xenografts were harvested, imaged, and weighed. Tumor tissues were used for subsequent detection by immunofluorescence and WB analysis.

### 2.10 Immunofluorescence

Slides of xenograft tissues were deparaffinized, rehydrated through an alcohol series, and subjected to heat-induced antigen retrieval using 0.01 M sodium citrate buffer (pH=6.0). Sections were incubated with primary antibodies against AKT (10176-2-AP, Proteintech; 1:100), p-AKT (#4060, Cell Signaling Technology; 1:100), HK2 (ab209847, Abcam; 1:100) and GLUT1 (ab115730, Abcam; 1:100) overnight at 4°C. After washing, the sections were incubated with Alexa Fluor 488 anti‐rabbit secondary antibody (A0423, Beyotime; 1:1000) for 1 h at room temperature. Nuclei were stained with DAPI (Beyotime), and immunofluorescence images were captured using a fluorescence microscope (Nikon, Tokyo, Japan).

### 2.11 Statistical analysis

Results of in vitro and in vivo experiments are recorded as the mean ± standard deviation (SD) of at least three independent experiments. SPSS v20 software (IBM) was used for statistical analyses. The Student's t-test with or without Welch's correction and ANOVA with post hoc Dunnet's test were used to compare the means of two and more than two groups, respectively. The correlation between PLEKHG2 expression and the prognosis of NSCLC patients was calculated by the log-rank test. A P-value <0.05 was used to indicate statistically significant differences.

## 3. Results

### 3.1 PLEKHG2 was upregulated in NSCLC and associated with poor prognosis

**Figure 1:**
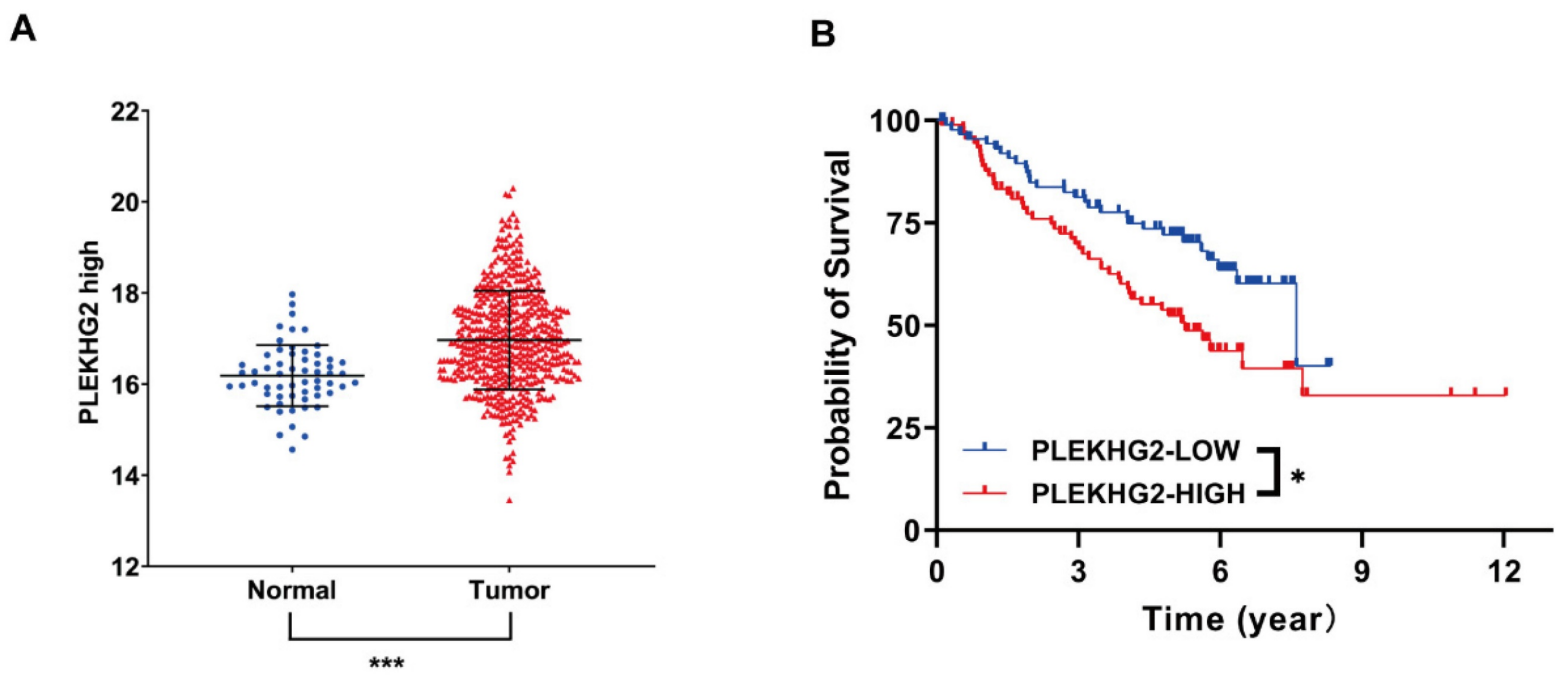
PLEKHG2 was upregulated in NSCLC and associated with poor prognosis. A: Comparison of RNA-SEQ data from TCGA-LUAD dataset to confirm the expression level of PLEKHG2 in NSCLC patients. The expression level of PLEKHG2 in NSCLC tumor tissues (n=526) was 1.05-fold higher than that in normal tissues (n=59). B: To further investigate the significance of PLEKHG2 elevation in tumor tissues, we analyzed the relationship between PLEKHG2 and prognosis in NSCLC. The median PLEKHG2 expression was used to group a total of 181 patients from GES50081. Comparisons revealed a poor prognosis in the group of NSCLC patients with higher PLEKHG2 expression (*:*P* < 0.05; ***: *P* < 0.001;).

RNA‐seq data from TCGA were used to evaluate PLEKHG2 expression in NSCLC. The results demonstrated that PLEKHG2 expression was significantly higher in NSCLC tissues (n=526) compared with in adjacent normal tissues (n=59) (*P*<0.001, Figure 1A). The GEO gene expression dataset GSE50081 (including 181 NSCLC samples) was selected to assess the relationship between PLEKHG2 mRNA expression and survival in NSCLC patients. The baseline characteristics of the patients from GSE50081 were provided in Supplemental Table 2. The expression of PLEKHG2 were associated with patients` smoking. The prognosis of NSCLC patients with high PLEKHG2 expression was worse than that of patients with low PLEKHG2 expression (*P* <0.05, Figure 1B).

**Figure 2:**
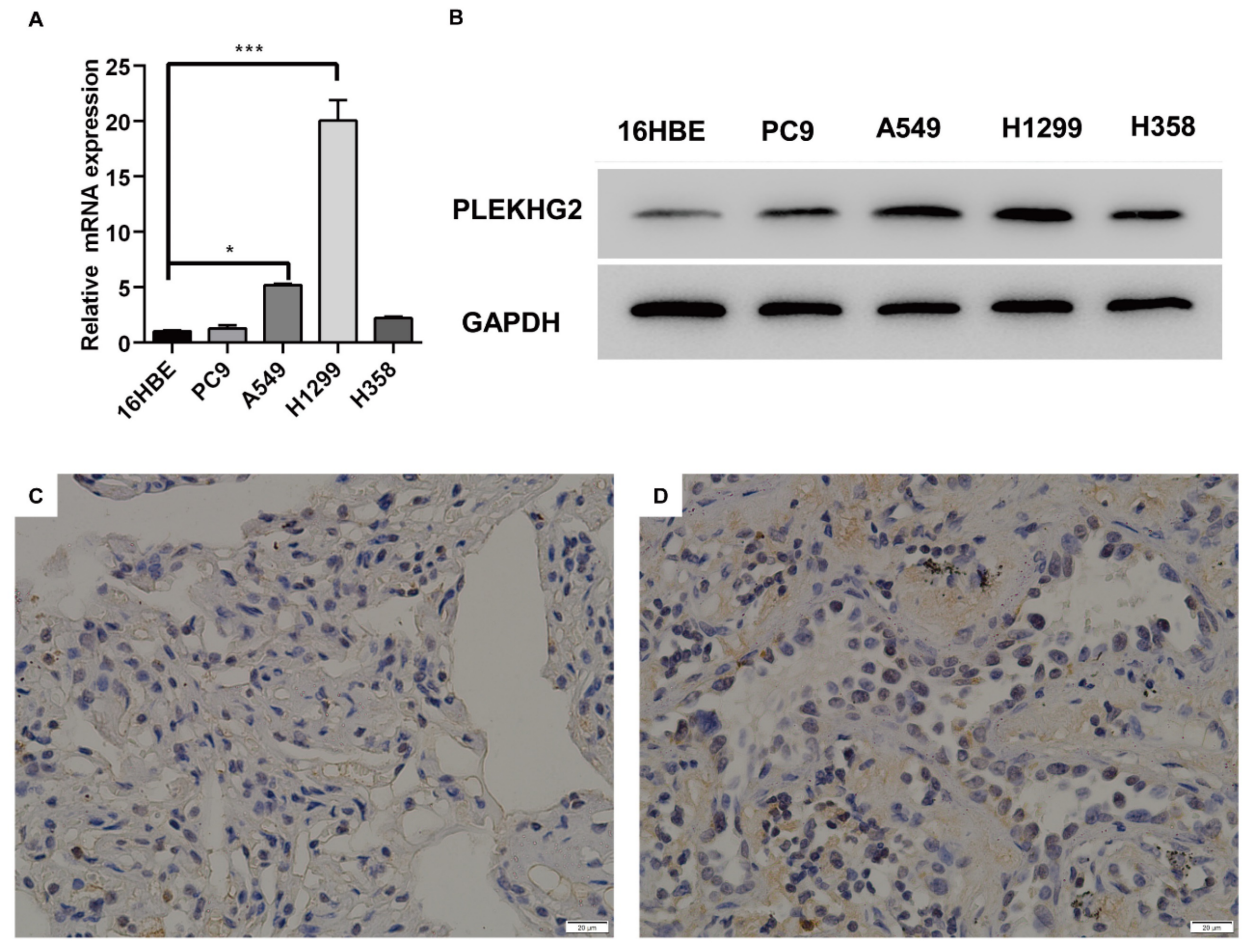
Validation of high expression of PLEKHG2 in NSCLC tumor tissues. Four NSCLC cell lines and one normal cell line were used to compare PLEKHG2 expression at the transcriptional (A) and protein (B) levels, respectively. PLEKHG2 was significantly increased at both the transcriptional and protein levels in the NSCLC cell lines. C, D: the NSCLC patients' tumor tissues (C) and paracancerous tissues (D) were further used for confirmation. The expression level of PLEKHG2 was higher in tumor tissues than in paracancerous tissues by IHC (*:*P* < 0.05; ***: *P* < 0.001;).

To further validate PLEKHG2 expression in NSCLC, four NSCLC cell lines were selected for comparison with the human normal bronchial epithelial cell line. Among the four tumor cell lines, mRNA expression levels of PLEKHG2 were 5.16- and 20.04-fold higher in A549 and H1299 cells, respectively, compared with 16HBE cells (*P* <0.05, Figure 2A). Meanwhile, PLEKHG2 protein levels were also found to be increased in all four tumor cell lines compared with 16HBE cells (Figure 2B). Furthermore, the difference in PLEKHG2 expression was verified by IHC using five paired NSCLC and corresponding paracancerous tissues. PLEKHG2 was barely detected in paraneoplastic tissues (Figure 2C), while intracellular PLEKHG2 was clearly seen in the nucleoplasm and cytosol of tumor tissues (Figure 2D). All patients' IHC images were shown in the Supplemental Figure 1. In summary, PLEKHG2 showed broadly upregulated expression in tumor tissues and was correlated with poor prognosis in NSCLC patients.

### 3.2 PLEKHG2 promoted glycolysis in NSCLC cells in vitro

**Figure 3:**
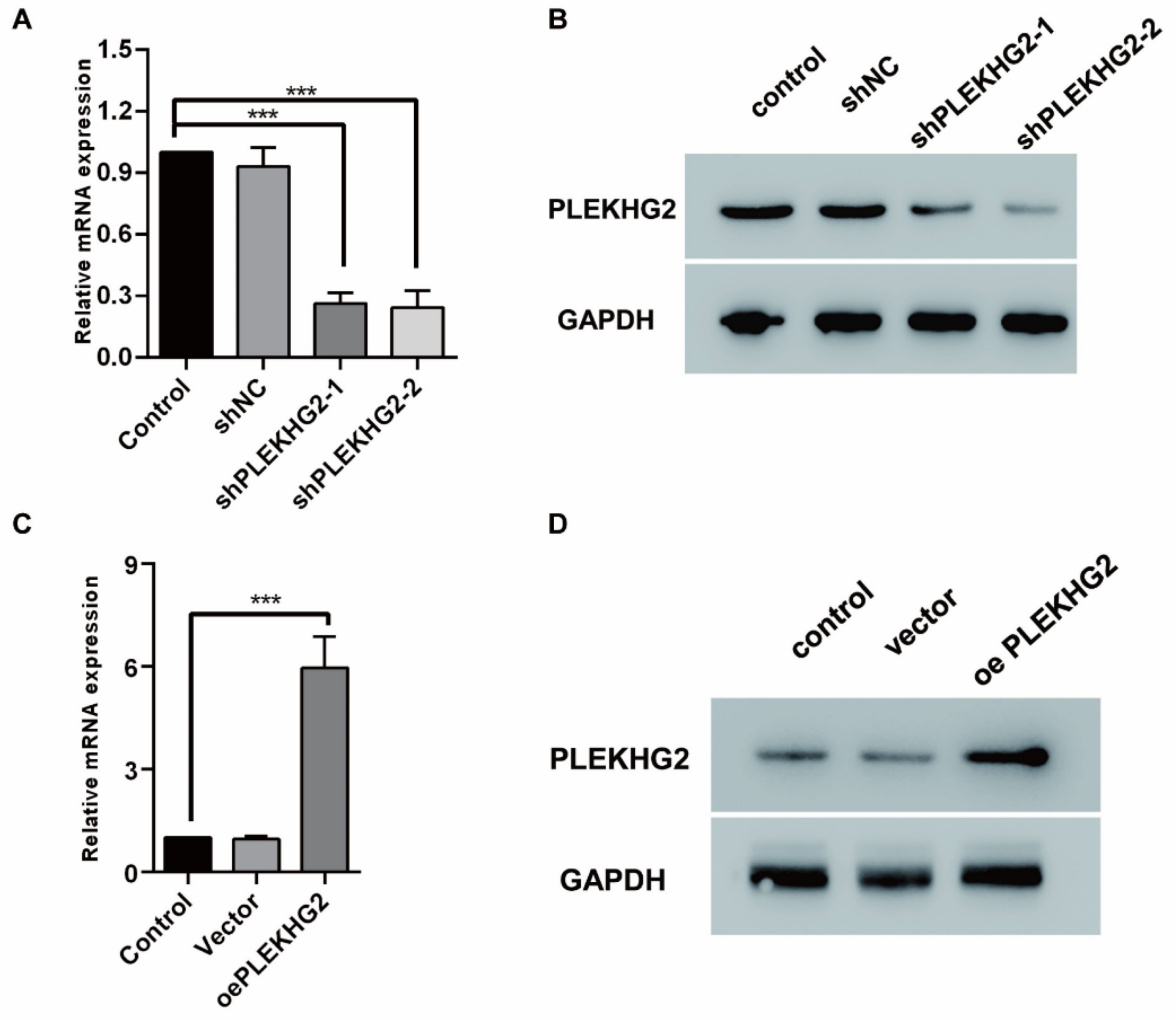
Construction of PLEKHG2 silencing and overexpression cell lines. To further investigate the function of PLEKHG2, we constructed PLEKHG2 silencing and overexpressing cell lines for subsequent functional assays, respectively. After transfection of shRNA, both transcription (A) and protein (B) levels of PLEKHG2 were significantly downregulated in H1299. And in PC9 transfected with a plasmid overexpressing PLEKHG2, PLEKHG2 was also successfully increased at the transcriptional (C) and protein (D) levels (***: *P* < 0.001;).

To explore the biological function of PLEKHG2, we used lentiviral-infected NSCLC cell lines with upregulated or downregulated PLEKHG2 expression. H1299 cells, which had the highest PLEKHG2 expression among the four NSCLC cell lines, were transduced with shRNAs. As shown in Figure 3A and 3B, both shRNAs caused a remarkable decrease in the mRNA and protein expression of PLEKHG2. In contrast, PC9 cells, which had the lowest PLEKHG2 expression among the four NSCLC cell lines, were transduced with the overexpressed vector. As shown in Figure 3C and 3D, PLEKHG2 expression was significantly upregulated in oePLEKHG2 cells.

**Figure 4:**
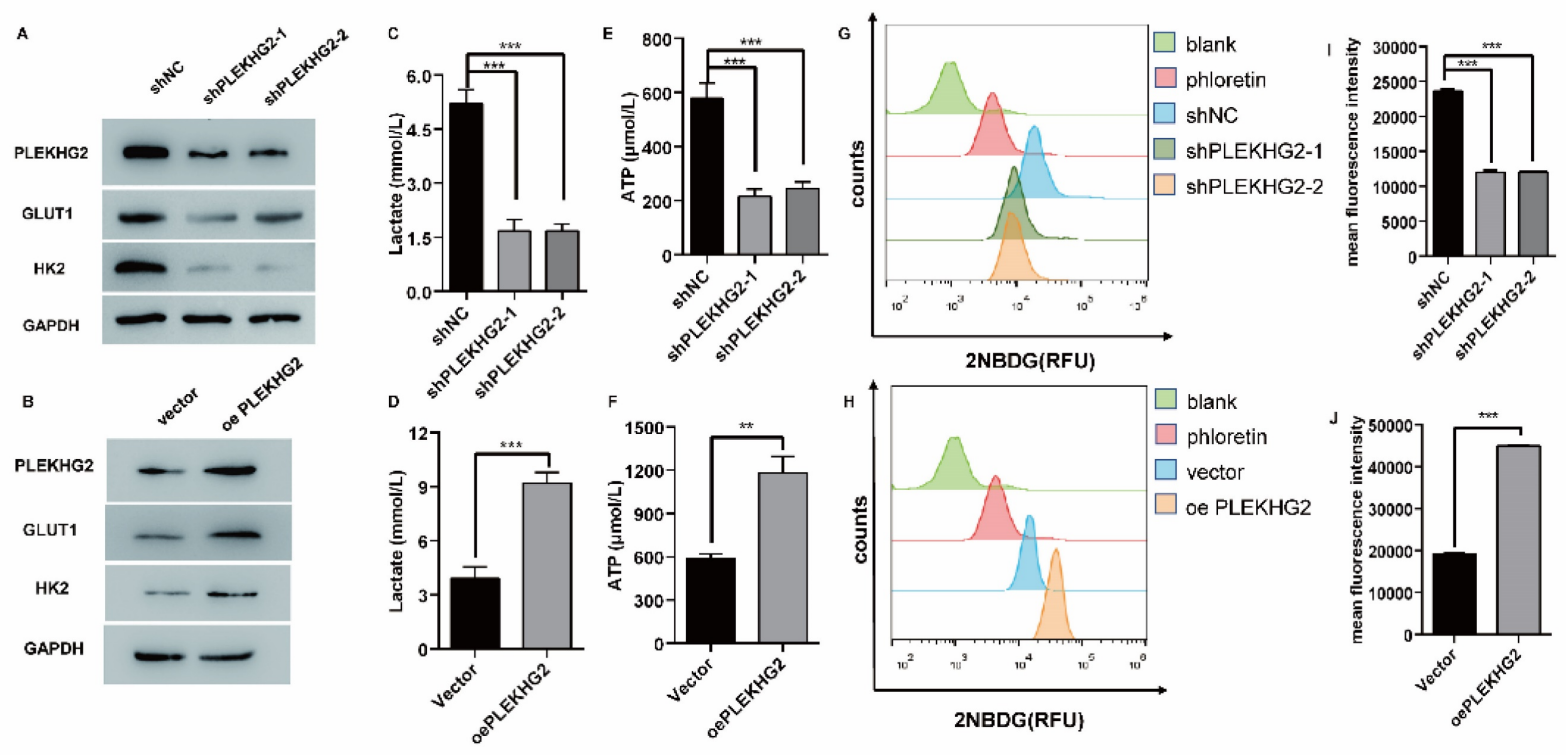
PLEKHG2 promoted glycolysis in NSCLC cells in vitro. The role of PLEKHG2 in glycolysis was first confirmed by in vitro assays. As the expression levels of PLEKHG2 were downregulated (A) or upregulated (B), the expression levels of GLUT1 and HK2 showed consistent changes. The decrease of lactate (C) and ATP (E) secreted by the cells and the impaired uptake of glucose (G, I) were detected when PLEKHG2 expression was inhibited. In contrast, there were increased lactate (D) and ATP (F) secretion and significantly higher glucose uptake (H, J) in the overexpressing PLEKHG2 cell line (***: *P* < 0.001;)

Furthermore, the relationship between PLEKHG2 expression and glycolysis in NSCLC cells was determined by detecting GLUT1 and HK2 expression in the PLEKHG2 knockdown and overexpression cells. The results demonstrated that GLUT1 and HK2 expression were positively correlated with PLEKHG2 expression (Figure 4A, B). To verify the function of PLEKHG2, lactate and ATP were detected in PLEKHG2-modified NSCLC cell lines. Compared with the unmodified NSCLC cell lines, lactate production was significantly decreased by 0.31- to 0.32-fold in cells with PLEKHG2 knockdown and increased by 2.01-fold in cells with PLEKHG2 overexpression (Figure 4C, D). ATP production showed the same trend. Compared with the unmodified cell lines, ATP levels in the shPLEKHG2-1, shPLEKHG2-2, and oePLEKHG2 groups were decreased by 0.31-, 0.32-fold, and increased by 2.35-fold, respectively. (Figure 4E, F). Additionally, we evaluated the glucose uptake capacity of NSCLC cells after PLEKHG2 knockdown or overexpression by detecting 2NDB-labeled glucose. The glucose uptake capacity of H1299 cells was significantly reduced after PLEKHG2 knockdown (Figure 4G, H) In contrast, PLEKHG2 overexpression increased glucose uptake in PC9 cells (Figure 4I, J). Collectively, these results demonstrated that PLEKHG2 promotes glycolysis in NSCLC cells.

### 3.3 PLEKHG2 regulated glycolysis via activating the PI3K/AKT pathway in NSCLC cells

**Figure 5:**
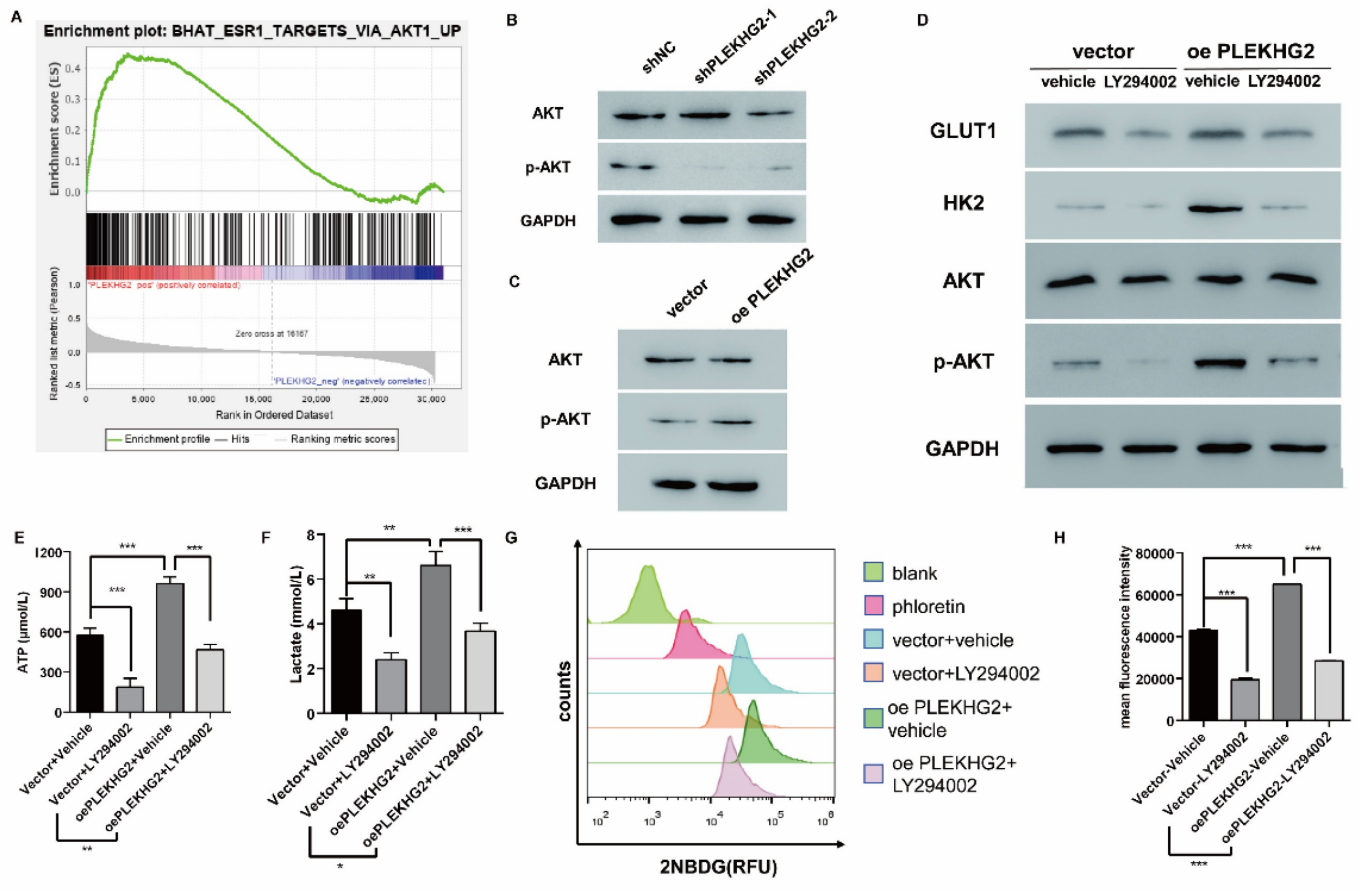
PLEKHG2 regulated glycolysis via activating the PI3K/AKT pathway in NSCLC cells. To investigate the mechanism of the PLEKHG2, we first used GSEA to investigate the PLEKHG2-enriched pathway. GSEA analysis suggested that PLEKHG2 potentially acted by regulating PI3K/AKT pathway activation (A). As PLEKHG2 expression was downregulated (B) or upregulated (C), AKT phosphorylation was also significantly inhibited or activated. LY294002, an inhibitor of the PI3K/AKT pathway, was used to confirm that PLEKHG2 promotes glycolysis by regulating the PI3K/AKT pathway. GLUT1, HK2 and AKT phosphorylation were all significantly inhibited in PC9 cells overexpressing PLEKHG2 after LY294002 supplementation, but were still higher than in PC9 cells without overexpressing PLEKHG2 (D). Also, the ATP (E), lactate (F) secretion and glucose uptake (G, H) of PC9 cells were all significantly inhibited by LY294002 supplementation. However, overexpression of PLEKHG2 antagonized the inhibitory effect of LY294002 (*:*P* < 0.05; **: *P* < 0.01; ***: *P* < 0.001;).

To further explore the mechanism though which PLEKHG2 regulates glycolysis in NSCLC cells, GSEA was performed with TCGA-LUAD dataset to screen relative pathway enrichment of PLEKHG2. This analysis indicated that PLEKHG2 expression was closely associated with activation of the AKT pathway, a key pathway involved in cellular metabolism and glycolysis (Figure 5A). By detecting the phosphorylation level of AKT, we found that PLEKHG2 expression promoted AKT phosphorylation, thereby activating the PI3K/AKT pathway (Figure 5B, C). These results suggest that PLEKHG2 promotes glycolysis in NSCLC cells by activating the PI3K/AKT pathway.

The PI3K/AKT pathway inhibitor LY294002 was used to validate the relationship between PLEKHG2 and the AKT pathway in PC9 cells. LY294002 treatment abolished the increased AKT phosphorylation seen in oePLEKHG2 cells. Additionally, we found that GLUT1 and HK2 levels were significantly decreased when the AKT pathway was inhibited (Figure 5D). Inhibition of the PI3K/AKT pathway also affected glycolytic activity, with levels of ATP and lactate being decreased to 45.38% and 55.61%, respectively, in oePLEKHG2 PC9 cells treated with LY294002 (*P* <0.001, Figure 5E, F). Meanwhile, glucose uptake was also decreased to 43.63% in oePLEKHG2 PC9 cells treated with LY294002 (*P*<0.001, Figure 5G~J). However, the same suppression of glycolysis was also found in unmodified PC9 cells and was more prominent. Intracellular ATP (*P*<0.01) and lactate (*P*<0.05) levels and glucose uptake (*P*<0.001) were significantly higher in treated oePLEKHG2 cells than in unmodified PC9 cells with the same treatment. These data demonstrated that upregulating PLEKHG2 antagonized the inhibitory effect of LY294002. Thus, we suggested that PLEKHG2 promotes glycolysis in NSCLC cells by activating the PI3K/AKT pathway.

### 3.4 Knocking down PLEKHG2 inhibited NSCLC xenograft tumor growth in vivo

**Figure 6:**
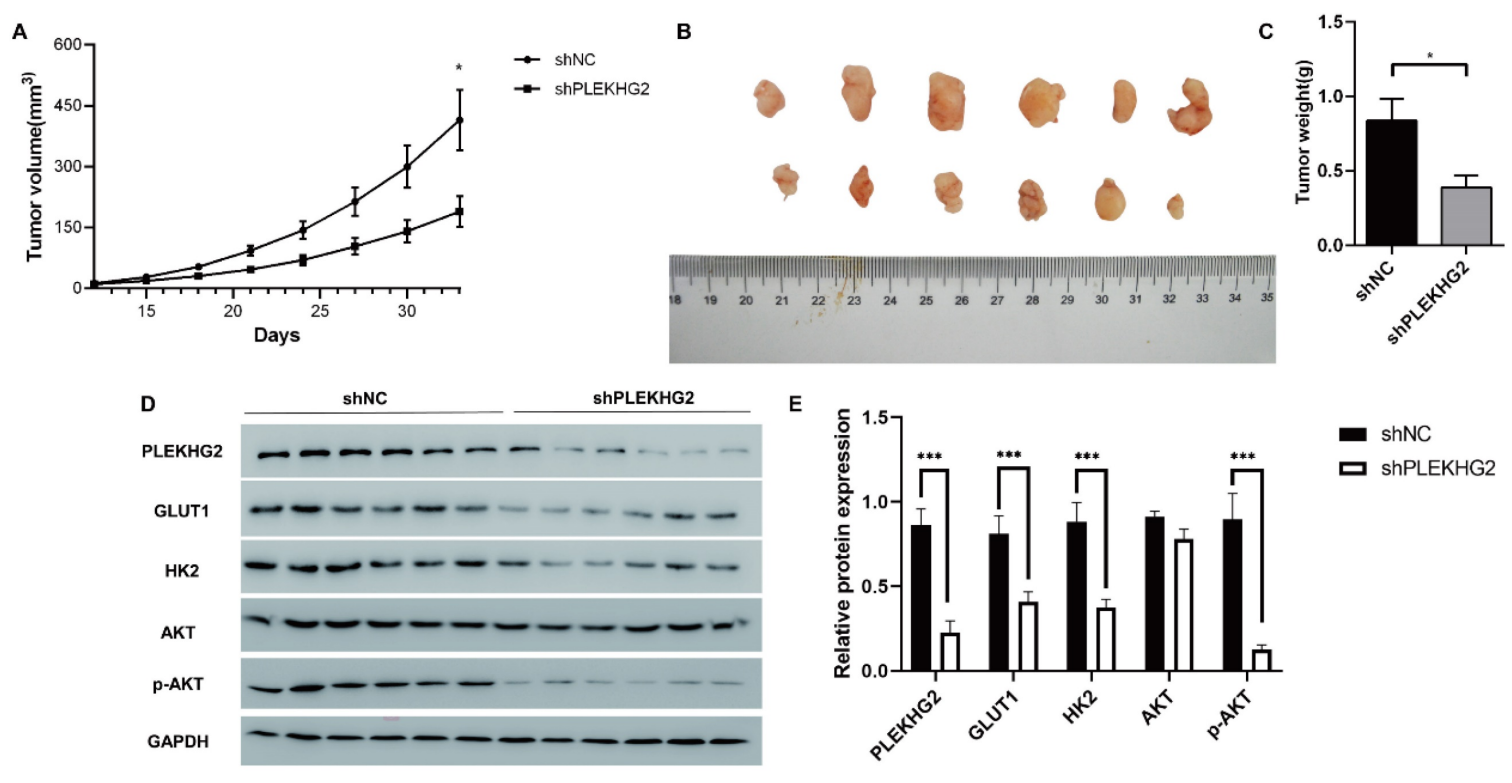
Knocking down PLEKHG2 inhibited NSCLC xenograft tumor growth in vivo. To further investigate the function of PLEKHG2 in vivo, we constructed a cell xenograft model by subcutaneous injection with H1299-silenced or unsilenced cells. The growth of tumors was significantly slowed down after PLEKHG2 inhibition (A). And tumor volume (B) and tumor weight (C) of mice in the PLEKHG2-silenced group were significantly lower than those in the control group at day 33 after injection. We also compared the activation of glycolysis and the PI3K/AKT pathway at different groups in vivo. intratumoral GLUT1, HK2, and AKT phosphorylation were significantly inhibited after PLEKHG2 inhibition (D, E) (*:*P* < 0.05; ***: *P* < 0.001;).

We next further verified the function of PLEKHG2 in the NSCLC xenograft model in vivo. As shown in Figure 6A, shPLEKHG2 tumors grew slower than shNC tumors (*P*<0.05). At 33 d post-implantation, tumor weights in the shPLEKHG2 group were reduced 46.51% compared with the shNC group (Figure 6B-E). Consistent with the in vitro results, PLEKHG2 knockdown remarkedly downregulated GLUT1, HK2, and p-AKT protein levels, while total AKT was unaffected (*P*<0.001, Figure 6F, G).

**Figure 7:**
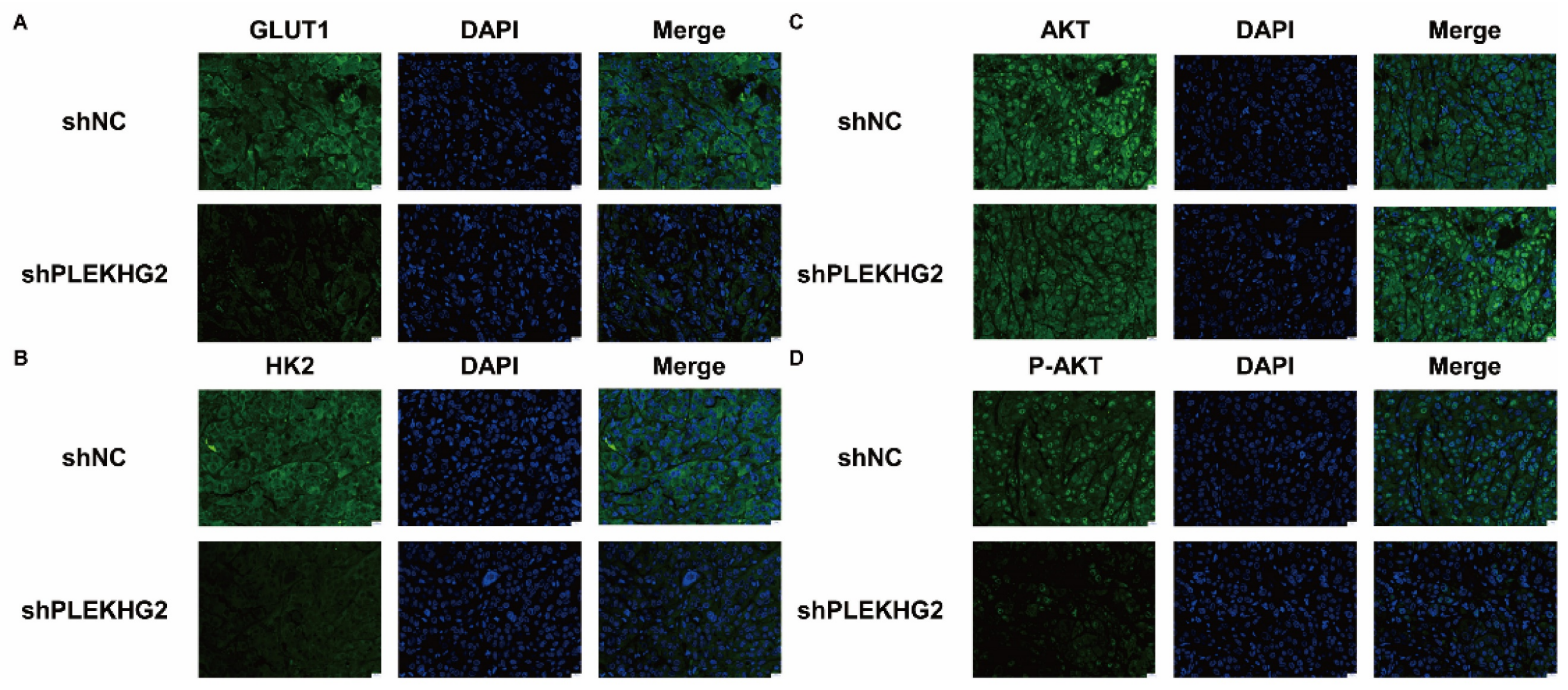
Decreased glycolysis in non-small cell lung cancer via suppression of PIK3-AKT pathway after silencing PLEKHG2. Further, we confirmed GLUT1 (A), HK2 (B) and AKT phosphorylation (C, D) of the tumor by immunofluorescence. GLUT1, HK2 and AKT phosphorylation were significantly inhibited after PLEKHG2 inhibition. Proteins were labeled by AF488 (green) and the nucleus was labeled by DAPI (blue).

Furthermore, we analyzed activation of the PI3K/AKT pathway and glycolytic markers by immunofluorescence. GLUT1 (Figure 7A) and HK2 (Figure 7B) staining was noticeable in the cytoplasm of H1299 cells, and the fluorescence intensity was significantly decreased in shPLEKHG2 cells. Meanwhile, AKT phosphorylation was also inhibited by shPLEKHG2 (Figure 7C, D). DAPI staining did not show any apparent changes in nuclear morphology, suggesting that silencing PLEKHG2 did not induced apoptosis in H1299 cells. In conclusion, knocking down PLEKHG2 inhibited the growth of NSCLC cells in vivo by reducing glycolysis via inhibiting the AKT pathway.

## 4. Discussion

Although the prognosis of NSCLC patients has been improving with continuing breakthroughs in research into driver genes and the availability of several new therapeutic options 15-17. However, rapid drug resistance due to tumor heterogeneity is also creating new challenges in NSCLC 18, 19. In this study, we focused on investigating the role of PLEKHG2 in NSCLC. There have been a few previous reports on PLEKHG2 in tumors, for example as early as in 2013, Shain et al. found that PLEKHG2 was likely among the oncogenes driving 19q13 amplification in pancreatic cancer^20^. However, a recent report by Liu et al. provided contrary evidence that PLEKHG2 upregulation improves drug sensitivity and patient survival in head and neck cancers^21^. These paradoxical results suggested that PLEKHG2 may have different roles in various cancer. Thus, we first confirmed the differential expression of PLEKHG2 in NSCLC. Bioinformatics analysis performed on the NSCLC dataset from GEO and TCGA in These paradoxical results suggest that PLEKHG2 may have different roles in various cancers. Thus, we first confirmed the differential expression of PLEKHG2 in NSCLC. Bioinformatics analysis performed on NSCLC datasets from GEO and TCGA indicated that PLEKHG2 expression was increased in tumor tissues compared with normal lung tissues and that high PLEKHG2 expression was associated with poor prognosis. Furthermore, we detected PLEKHG2 expression in NSCLC cell lines and tumor tissues from patients and confirmed that PLEKHG2 expression was upregulated in NSCLC. These results confirmed that PLEKHG2 was upregulated in NSCLC and associated with poor prognosis.

Next, we investigated the biological functional changes that resulted from the differential expression of PLEKHG2 in NSCLC. Considering that NSCLC presents high genetic and microenvironmental heterogeneity in actual clinical practice, we investigated whether the most common metabolic abnormality of tumors, glycolysis, was a potential functional target PLEKHG2^22^, ^23^. We demonstrated that GULT1 and HK2 protein levels were positively correlated with PLEKHG2 expression in tumor tissues both in vivo and in vitro. Overexpression and silencing of PLEKHG2 resulted in the up- and down-regulation of GLUT1 and HK2, respectively. GLUT1 is the facilitative glucose transporter that is responsible for constitutive or basal glucose uptake^24^. HK2 catalyzes the initial phosphorylation of hexose molecules 25. According to their important role in glycolysis, we then confirmed that upregulating PLEKHG2 promoted glycolysis in NSCLC cells. Moreover, glucose uptake and the primary products of glycolysis (lactate and ATP) were correlated with PLEKHG2 levels, which also supported this conclusion.

Activation of the PI3K/AKT pathway is the most common tumorigenic factor for reprograming cellular metabolism, as this enhances the activity of nutrient transport proteins and metabolic enzymes, thereby supporting the anabolic requirements of aberrant cell growth^26^. As the hallmark of PI3K/AKT activation, AKT phosphorylation has been shown to promote glycolysis 27. In this study, PLEKHG2 overexpression increased AKT phosphorylation and upregulated GLUT1 and HK2, as in previous reports 28, 29. The enhanced glycolysis in PLEKHG2-overexpressing PC9 cells was abolished by treatment with an AKT inhibitor, indicating that PLEKHG2 increased glycolysis via activating the PI3K/AKT pathway.

Recently, Liu et al. and Jayathirtha et al. also found that PLEKHG2 promotes glycolysis in head and neck cancer^21^ and breast cancer^30^, respectively. However, the effects of PLEKHG2 on tumorigenesis and prognosis were different in their reports. Certainly, the role of glycolysis in tumors is controversial. In general, the altered metabolic pattern of glycolysis produces metabolites, such as pyruvate and lactate, that create the material basis for the rapid proliferation of tumors 31. Moreover, the local accumulation of lactate also creates an acidic and hypoxic environment for tumors^32^, which has been shown to be associated with the disorderly angiogenesis typically found in advanced-staged tumors^33^, ^34^. Conversely, because of overactive and disrupted metabolism, glycolysis could increase the burden of tumor cells by creating abundant toxic products 35, 36. Thus, we further validated the effects of PLEKHG2 knockdown on NSCLC tumor growth in the H1299 xenograft tumor model. In vivo, PLEKHG2 knockdown inhibited the growth of H1299 xenograft tumors.

Restricted by laboratory conditions, we have only explored the role and mechanism of PLEKHG2 in NSCLC in immunodeficiency models. With the development of immunotherapy in recent years, the survival of NSCLC patients has been notably improved. Dramatic metabolic reprogramming of both cancer cells and immune cells occurs during both tumorigenesis and immunotherapy-based killing^37^, ^38^. Whether glycolysis promoted by PLEKHG2 upregulation affects the sensitivity of NSCLC to immunotherapy requires follow-up investigations in immunocompetent models or clinical studies.

In conclusion, we identified that PLEKHG2 is a key gene that affects the prognosis of NSCLC patients through bioinformatics and verified its ability to promote glycolysis in NSCLC cells through activation of the PI3K/AKT pathway in vivo and in vitro. Silencing PLEKHG2 inhibited the growth of H1299 xenografts in mice. This work demonstrates that PLEKHG2 is a potential biomarker of poor prognosis in NSCLC.

## Supplementary Material

Supplementary figure and tables.
