# Supplementary material for: PLEKHG2 Promotes NSCLC Cell Growth by Increasing Glycolysis via Activated PI3K/AKT Pathway
Source: J Cancer. 2023 Oct 30;14(18):3550–60. doi: 10.7150/jca.88857 (PMC10647195; doi:10.7150/jca.88857)

Supplementary Material

PLEKHG2 Promotes NSCLC Cell Growth By Increasing Glycolysis Via Activated PI3K/AKT Pathway

Yang Xia[1†], Xinyu Feng[2†], Yunye Ning[1], Zhenli Hu[1], Wei Zhang[1], Qianqian Chen[1], Jun Wang[1], Yuchao Dong[1*], Yang Lu[3*]

*** Correspondence:**
Yang Lu
toby08@163.com

Yuchao Dong
dongyc1020@126.com

Supplemental Table 1. Sequences of qPCR primers

primer		Sequence（5′→3′）
has-PLEKHG2	forward	AGGAAGGGCTGGAGATGGAT
	reverse	GAGAGGGTGGGAGTGCTAGA
has-GAPDH	forward	AATCCCATCACCATCTTC
	reverse	AGGCTGTTGTCATACTTC

Supplemental Table 2. Baseline characteristics of the patients from GSE50081

Variable		PLEKHG2-LOW	PLEKHG2-HIGH	*P*-value
Sample size		90	91	
Age (years)		68.77±9.68	68.79±9.05	0.97
Gender (%)	Male	47 (52.22)	51 (56.04)	0.72
	Female	43 (47.78)	40 (43.96)	
Smoking (%)	Never	20 (22.22)	4 (4.40)	＜0.001***
	Ex-smoker	47 (52.22)	53(58.24)	
	Current	23 (25.56)	34 (37.36)	
Stage (%)	Ia	31 (34.44)	17 (18.68)	0.16
	Ib	35 (38.89)	44 (48.35)	
	IIa	3 (3.33)	6 (6.59)	
	IIb	21 (23.33)	24 (26.37)	
Outcome (%)	Alive	61 (67.78)	45 (49.45)	0.017*
	Death	29 (32.22)	46 (50.55)	

*:$P < 0.05$; ***: $P < 0.001$;

Supplemental Table 3. The clinical characteristics of the patients for IHC

Variable	Paient1	Paient2	Paient3	Paient4	Paient5
Age (years)	68	38	65	66	69
Gender	Female	Female	Female	male	Female
BMI	21	23	25.5	27.2	27.1
Smoking	Never	Never	Never	Never	Current
hypertension	No	No	No	Yes	Yes
diabetes	No	No	No	No	Yes
Stage	Ia	Ia	Ia	Ia	Ia

Supplemental Figure 1. Validation of high expression of PLEKHG2 in NSCLC tumor tissues by IHC

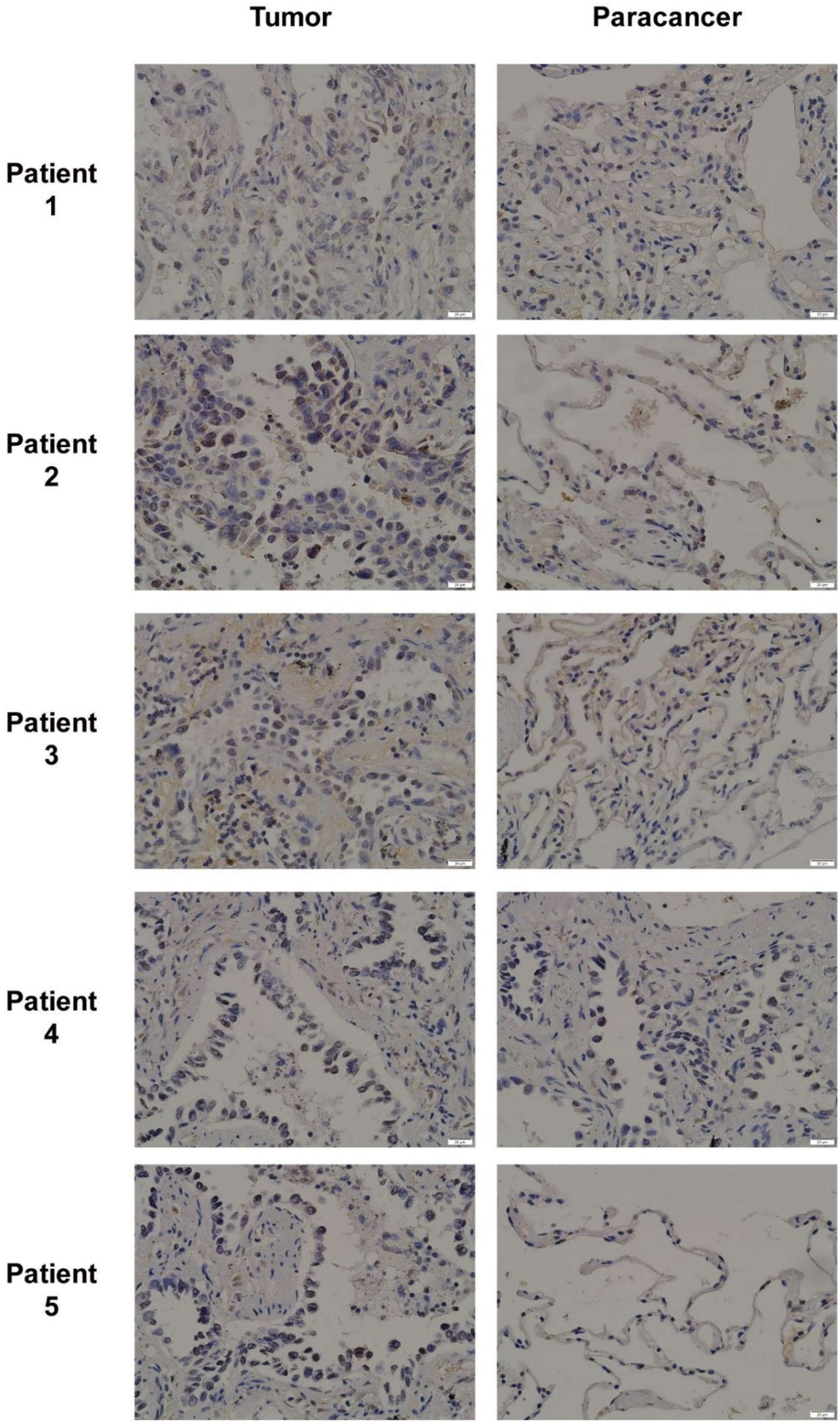

Supplement: Supplementary file 1 — Supplementary figure and tables. [file jcav14p3550s1.pdf]
